# Editorial: Molecular interactions and control strategies for *Botrytis cinerea* in crop production

**DOI:** 10.3389/fpls.2025.1624296

**Published:** 2025-05-26

**Authors:** Paulo Canessa, Simone Ferrari

**Affiliations:** ^1^ Centro de Biotecnologia Vegetal, Facultad de Ciencias de la Vida, Universidad Andres Bello and ANID–Millennium Science Initiative–Millennium Institute for Integrative Biology (iBIO), Santiago, Chile; ^2^ Department of Biology and Biotechnologies “Charles Darwin”, Sapienza University of Rome, Rome, Italy

**Keywords:** *Botrytis cinerea*, plant-pathogen interactions, plant immunity, biocontrol, gray mold

*Botrytis cinerea* is a well-known fungal phytopathogen that is responsible for gray mold in a wide variety of crops *–* from economically important tomatoes and grapes to widely adopted plant models such as *Arabidopsis thaliana* (Chen et al., 2023). It has been estimated that the economic losses associated with the adverse action of *B. cinerea* are approximately 100 billion USD per year globally (Dean et al., 2012). Paradoxically, under very distinct environmental conditions, this infamous pathogen can significantly increase the value of agricultural produce: this is the case of grape berries that undergo the so-called noble rot, enabling the production of *Sauternes* wines. Nonetheless, *B. cinerea* is a typical necrotrophic phytopathogenic fungus (Van Baarlen et al., 2007) that causes massive host cell death to facilitate colonization (Bi et al., 2023).

Over the past two decades, *B. cinerea* has become a model organism for studying fungal necrotrophs. In fact, it has been listed as the most scientifically and economically significant fungus of its kind (Dean et al., 2012). *B. cinerea* has benefited from recent advances in metabolomics, genomics, and transcriptomics — including the availability of multiple sequenced genomes, an exquisitely sequenced reference genome (Van Kan et al., 2017), and advanced tools for molecular visualization and genetic modification (Schumacher, 2012; Leisen et al., 2020) — providing exhaustive insights into the molecular interactions with its hosts. Despite these advances, the mechanisms of fungal virulence and defense responses orchestrated by the plant remain far from being fully deciphered. Understanding these mechanisms might contribute to devising strategies to enhance plant defenses or circumvent fungal virulence. In this Research Topic, readers will find articles investigating the molecular and physiological mechanisms underlying the interaction of *B. cinerea* with different host plants. These articles highlight the environmental and genetic/epigenetic context in which this interaction occurs.

By harnessing beneficial microbes and employing *Solanum lycopersicum* as a plant host, Ajijah et al. explored several beneficial bacteria as natural biocontrol antagonists. *Pseudomonas protegens* ML15 exhibits a direct antifungal activity, secretes a battery of secondary metabolites, and stimulates host defense responses in tomatoes. Notably, bacterial culture supernatants were able to significantly decrease the infection. This study illustrates the promising work being carried out today by different groups (Olivares-Yañez et al., 2025) to understand the biocontrol capabilities of different microorganisms, with the ultimate goal of creating new tools for the sustainable protection of different crops.

While beneficial microbes can directly enhance host protection (Ajijah et al.), the plant genetic architecture can either amplify or diminish defenses. Plant innate immunity triggered by microbial and plant-derived elicitors is one of the most important lines of defense against pathogens. Chitin-triggered immunity induces resistance to subsequent infection with *B. cinerea* (Giovannoni et al., 2021). In Arabidopsis, the CHITIN ELICITOR RECEPTOR KINASE 1 (CERK1) protein and related proteins mediate the perception and signalling of chitin oligosaccharides (Miya et al., 2007; Wan et al., 2008; Liu et al., 2012). CERK1 in turn phosphorylates downstream receptor-like cytoplasmic kinases to regulate immune responses (Zhang et al., 2010; Yamada et al., 2016; Liu et al., 2018). Despite our detailed knowledge of chitin perception and transduction, downstream signaling elements involved in chitin-mediated resistance to *B. cinerea* are only partially characterized. Chen et al. found that the Arabidopsis leucine-rich repeat receptor-like kinase ZYGOTIC ARREST 1 (ZAR1) interacts with dephosphorylated CERK1 and negatively contributes to resistance against *B. cinerea* independent of early chitin-triggered responses such as MAP kinase activation and reactive oxygen species accumulation.

Activation of Pattern-Triggered Immunity (PTI) is the first tier of the plant’s innate immune system. Upon pathogen entry, PTI can activate multilayered defense responses with varied effectiveness against *B. cinerea*. However, the outcome of the interaction between the fungus and its host varies greatly, depending on their genotypes and the environmental conditions. One striking example of this variability is the “noble rot” phenomenon, which is a latent form of infection that occurs in grape berries under peculiar microclimatic conditions characterized by dry and sunny days and humid nights (Ribéreau-Gayon et al., 1980; Magyar, 2011; Vannini and Chilosi, 2013). Noble rot results in the rapid withering of the grape berry, which is required to produce famous sweet white *Sauternes* wines. In sharp contrast, under continuous mild wet weather (typically the same laboratory conditions used to study *B. cinerea* virulence), the infection results in gray rot and the loss of the berry (Williamson et al., 2007). A transcriptomic analysis revealed that the so-called noble rot phase exhibits significant differences from the other two stages (Váczy et al.). This study shows that the initial stages of infection reflect a virulent fungus-plant interaction, regardless of whether the outcome is gray or noble rot. However, paradoxically, expression of host defense-related genes is suppressed during the noble rot stage, suggesting that the plant is not actively defending itself against *B. cinerea* and that the host and the fungus have reached an equilibrium.

Rounding out the picture portrayed in this Research Topic, emerging evidence indicates that epigenetic mechanisms also dictate how strongly plants resist pathogens. Epigenetic control of gene expression is crucial for all aspects of plant biology, including host-microbe interactions (Hannan Parker et al., 2022). However, few studies have focused on the epigenetic regulation of host responses to *B. cinerea* infection. The same is valid for regulating fungal virulence responses (Miao et al., 2022). For example, chromatin modifications appear to modulate the expression of the tomato transcription factor SlyWRKY75, which in turn regulates the defense-related jasmonate (JA)-dependent pathway (López-Galiano et al., 2018). Liang et al. reported on the characterization of watermelon ClMBD2, ClMBD3, and ClMBD5 proteins, which encode Methyl-CpG-Binding Domain (MBD) proteins, which in turn are known to act as transcriptional repressors associated with methylated DNA. Their overexpression in Arabidopsis reduces resistance against *B. cinerea* and downregulates the expression of *AtPDF1.2*, suggesting that they negatively regulate JA responses. In contrast, the overexpression of ClMBD5 led to increased resistance against *Pseudomonas syringae.*

As the quest for plant targets that can be modified by gene editing to create varieties resistant to *B. cinerea* continues, this Research Topic also exemplifies a seldom observed dimension to our understanding of how plants fine-tune immunity: some genes that may be essential for developmental processes can incidentally or simultaneously downregulate defense. As indicated here, these targets must be carefully evaluated since it is possible to generate difficult-to-anticipate effects, including diminished resistance or increased defense, depending on the pathogen.
